# Follicular lymphoma or diffuse large B-cell lymphoma: a population based analysis of epidemiological and health economic aspects in Germany

**DOI:** 10.1007/s00277-025-06592-8

**Published:** 2025-09-02

**Authors:** Karin Berger, Bernhard Moertl, Michael von Bergwelt-Baildon, Dominik Obermüller, Dorota Pawlowska-Phelan, Martin Dreyling

**Affiliations:** 1 Department of Medicine III, University Hospital, LMU Munich, Munich, Germany; 2 InGef - Institute for Applied Health Research Berlin GmbH, Berlin, Germany

**Keywords:** Claims data analysis, Lymphoma, German, Healthcare resource utilization, Costs, Real world, DLBCL, FL, CAR-T

## Abstract

Contemporary information on epidemiology, healthcare resource utilization (HCRU), costs and clinical outcomes in routine care is essential for value-based decision-making. However, such information remains limited for follicular lymphoma (FL) and diffuse large B-cell lymphoma (DLBCL) in Germany. This study addresses these gaps. This retrospective cross-sectional cost-of-illness study analyzed anonymized statutory health insurance claims data (2015–2020). FL Grade I-IIIa (ICD-10: C82.0-C82.3), DLBCL (ICD-10: C83.3) patients were identified via inpatient or outpatient ICD coding. Elixhauser and Charlson Comorbidity Indices were used to describe the general comorbidity burden. FL prevalence increased from 26 to 32 per 100,000 insured persons (*n* = 837 to 1,028), DLBCL prevalence rose from 37 to 45 per 100,000 (*n* = 1,205 to 1,437). Mean age (FL: 67.0 ± 13; DLBCL: 68.6 ± 13.6) and sex distribution (FL: 50% female; DLBCL: 44% female) remained stable 2015–2020. Mean Charlson Comorbidity Index 4.1 ± 2.4 (FL), 4.8 ± 2.7 (DLBCL), mean Elixhauser 5.2 ± 3.0 (FL), 6.1 ± 3.3 (DLBCL). Hospitalization rates: 64% of FL patients (2.0 ± 2.3 admissions, 21 ± 44.7 days/year); 78% of DLBCL patients (2.9 ± 3.1 admissions, 29 ± 47.5 days/year). Mean annual costs per patient in third-party payers perspective were €15,258 (FL), €23,455 (DLBCL). Post-SCT 12-month costs were €46,270 (FL), €56,558 (DLBCL) for autologous-SCT, and €161,662 for allogeneic-SCT (DLBCL only). Rising prevalence calls for ongoing real-world assessment of HCRU and costs. This study supplements limited evidence, highlighting significant economic impact. While health insurance data offer valuable insights, their lack of clinical details necessitates integration with other data sources. Several initiatives are building data spaces to enhance evidence generation; meanwhile, analyses based on single data sources remain valuable to inform practice and policy.

**Supplementary Information:**

The online version contains supplementary material available at 10.1007/s00277-025-06592-8.

## Introduction

Hematological malignancies include a heterogeneous group of lymphomas, multiple myelomas, and leukemias [1]. They differ according to cell type, clinical and molecular characteristics, prognosis, and treatment options [2, 3]. Follicular (FL) and diffuse large B-cell lymphomas (DLBCLs) are the most common subtypes of malignant lymphomas (MLs) [4, 5]. Both entities arise from B cells, are heterogeneous, and can develop aggressive grades.

For DLBCL first-line therapy, cure rates of approximately 60–80% have been reported for patients with DLBCL [6, 7]. In contrast, there is no curative setting for FL patients, and the overall treatment aim is long-term remission [2, 8]. Watch-and-wait is an established approach for FL, whereas DLBCL progresses rapidly without any treatment. International publications have shown reduced survival rates for patients with relapsed or refractory disease (r/r) in both entities [6, 9–13].

A wide variety of treatment concepts is applied to treat r/r patients, depending on age, general condition, response rate, timing, and other clinical indicators [14]. Various chemotherapy regimens, immunotherapies, radiotherapies, stem cell transplantations (SCTs), and targeted options are recommended by national FL and DLBCL guidelines [15, 16]. In recent years, innovative therapies for r/r patients have been approved by the European Medicines Agency (EMA), e.g. bispecific antibodies or therapies with chimeric antigen receptor (CAR) T-cells [10, 17, 18]. In addition to the clinical aspects and benefits, the economic consequences of these novel treatment options for third-party payers are not entirely known. Recently, the first German budget impact analysis of CAR T-cell therapy for adult patients with r/r DLBCL estimated a budget impact of €39 to €166 million for statutory health insurers [19].

For a comprehensive understanding of the economic delta between both standard and innovative treatment options, data from routine care is required. These data include detailed information on patient characteristics, treatment patterns, resource use, costs, and outcomes. Such data are crucial not only for optimizing patient care but also for assessing the economic impact of these treatments, enabling healthcare providers and policymakers to make informed decisions about resource allocation and treatment strategies. However, comprehensive trans-sectoral longitudinal information on FL and DLBCL patient treatment journeys, treatment patterns, and resource consumption is still limited in many places. This evidence gap presents significant challenges for demonstrating value, making informed decisions, and developing future care models for responsible stakeholders. The analysis was conducted to partially address this evidence gap by generating additional information on FL and DLBCL care in Germany. This is an initial step that not only fills some of the data voids but also aims to stimulate discussion and catalyze further research and policy development in this critical area of healthcare.

## Materials and methods

### Study Design

This analysis is a retrospective cost-of-illness study based on the InGef research database. The observation period covers the years from 2015 to 2020. Depending on the respective research question, individual analyses were conducted as cross-sectional analyses. Subgroup analyses on stem cell-transplanted patients were conducted as longitudinal analyses. Variables, such as demographic and clinical characteristics, diagnostic methods, medical therapies, procedures, adverse events, resource utilization, costs, and mortality, were considered separately for prevalent patients of both entities on an annual basis. The analysis was pre-specified as a prevalence-based cost-of-illness snapshot, focusing on absolute annual burden within the study cohorts; it was not intended to estimate incremental or excess outcomes versus matched general population controls. Given the nature of anonymized statutory health insurance claims, permissible look-back windows and data-use approvals do not allow reliable incident-case ascertainment (i.e., exclusion of prior diagnoses/treatments outside the observation window). Long treatment-free intervals after first-line immunochemotherapy (e.g., R-CHOP) can under limited look-back later relapse and appear as ‘new’ cases. We therefore pre-specified prevalence-based, calendar-year cohorts; newly diagnosed (incident) patients are included in these cohorts but are not labeled separately.

## DATA SOURCE

The InGef database contains anonymized, longitudinal statutory health insurance (SHI) data of approximately 9million individuals who are insured by one of more than 50 German health insurance companies included in this database. For this analysis, an annual representative sample of approximately 3.25million insured adult persons was used, which is representative of the age and sex distributions of the German adult population [20]. The total observation period available is limited to six years.

The InGef research database contains sociodemographic information such as sex, age, and region of residence. In addition, detailed information on inpatient and outpatient care was obtained from the database. The inpatient data include the date of admission, the date of discharge, and diagnostic and therapeutic information (OPS-level), with exact dates and diagnoses. The outpatient data also comprise diagnostic and therapeutic information. The outpatient prescription data contain information (type and quantity) at the ATC level. All diagnoses are coded according to the German Modification of the International Classification of Diseases, 10th Revision (ICD-10-GM).

All insured individual data in the InGef database are anonymized and are no longer social data in the sense of §67 para. 2 SGB X in combination with Art. 4 No. 1 DSGVO, and their use for scientific research purposes is compliant with German laws; accordingly, no further permission from an ethics committee is needed. The analysis follows the recommendations of Good Epidemiological Practice (GEP) and Good Practice Secondary Data Analysis (GPS).

### Case definition

Follicular lymphoma Grade I-IIIa (ICD-10: C82.0–C82.3) and diffuse large B-cell lymphoma (ICD-10: C83.3) were identified by ICD coding as inpatient main or secondary diagnoses and/or two outpatient diagnoses in different quarters of the respective analysis year (M2Q criterion). If an initial outpatient diagnosis is followed by an inpatient diagnosis in another quarter, the outpatient diagnosis defines the index date. If there was an outpatient and an inpatient diagnosis in the same quarter, the date of the inpatient diagnosis was defined as the index date. Patients with any additional malignancy coding (any other ICD-10-C-coding) during the observational period were excluded from the analysis.

### Study variables

Patient characteristics were assessed according to the individual index year during the observation period. Sociodemographic variables included age and sex. The Elixhauser and Charlson Comorbidity Scores were used to describe the general comorbidity burden. For comorbidity scoring, entity-relevant ICD codes (e.g., lymphoma) were considered [21, 22]. In addition, the specific comorbidity burden was assessed, identifying and describing the five most common documented comorbidities. Medical variables and measures included diagnostics (ICD-10 codes), outpatient medication (ATC-L codes), relevant inpatient and outpatient procedures (OPS codes), side effects (ICD-10 codes; e.g., sepsis, thrombosis) and outcomes in terms of mortality. In German claims, OPS procedure codes exist for both inpatient and outpatient services. With respect to systemic treatments, the outpatient sector additionally records prescription-level pharmacy claims at ATC L-code (product) level, which allows substance-level analyses (reported in the Supplement). The inpatient sector bills systemic therapies via aggregated OPS procedure codes that do not capture exact drug compositions or dosing; therefore, regimen-level attribution is not feasible for inpatient care. Because coding granularity differs by setting, outpatient and inpatient treatment figures are not directly additive; we report them separately to avoid misclassification. Economic variables comprised inpatient stay and costs in third-party payer perspective.

### Subgroup analysis of patients who underwent stem-cell transplantation

Identification was performed via OPS coding. Autologous stem cell transplantations (auto-SCT) with the OPS codes 5-411.0, 8-805.0 and allogeneic stem cell transpantations (allo-SCT) with the documented OPS codings 5-411.2–5-411.5, 8-805.2–8-805.5. SCT subgroup analyses were carried out for 6, 12, 24 months after treatment. The following measures were assessed separately within the available individual follow-up period: (1) Patient characteristics and comorbidities at the time of SCT therapy. (2) Number and duration of hospitalizations for patients with 6, 12, 24 months follow-up after therapy. 3) Costs of transplant at 6, 12, 24 months after SCT coding. 4) Mortality using Kaplan‒Meier curves at 6, 12, 24 months after therapy. Survival curves are displayed up to 24 months, consistent with the pre-specified analysis horizons and the 2015–2020 observation window. Only patients with complete follow-up for the displayed horizon are included.

## RESULTS

### Cohort description – patient characteristics and comorbidities

Between 2015 and 2020, a mean number of 956 prevalent FL patients and 1,362 prevalent DLBCL patients were identified. The prevalence of patients with FL increased from 26 patients per 100,000 insured individuals in 2015 up to 32 per 100,000 in 2020. In the same period, the prevalence of patients with DLBCL rose from 37 to 45 patients per 100,000 insured individuals (Table 1).

**Table 1:** Absolute number** (n) **of prevalent patients per year and per 100,000 insured individuals per year patient characteristics and comorbidity scores of FL and DLBCL cohort remained relatively stable during the observation period 2015–2020. As examples, both cohorts from the year 2020 are shown in Table 2. More details are presented in the supplement Tables 1 - 3

	FL			DLBCL		
Number of individuals per year; n absolute (n per 100,000)	n		per 100,000	n		per 100,000
2015	837	26		1,205	37	
2016	901	27		1,324	40	
2017	927	28		1,349	41	
2018	1,012	31		1,414	44	
2019	1,030	32		1,445	44	
2020	1,028	32		1,437	45	

**Table 2:** Patient characteristics of the prevalent FL/DLBCL cohort in the year 2020

	2020’ FL cohort		2020’ DLBCL cohort	
Age (in years)	mean	SD	mean	SD
	68	13	69	14
	Median	Min-Max	Median	Min-Max
	69	21–98	71	19–97
Age groups (in years)	n	%	n	%
18–39	27	3	53	4
40–59	210	22	262	19
60–80	596	62	796	58
> 80	123	13	252	18
Sex	n	%	n	%
Male	471	49	758	56
Female	485	51	605	44
Comorbidities scores	mean	SD	mean	SD
Charlson Comorbidity Index	4,2	2,4	4,7	2,7
Elixhauser Comorbidity Index	5,3	3,0	5,9	3,3
	Median	Min-Max	Median	Min-Max
Charlson Comorbidity Index	3,0	2,0–13,0	4,0	2,0–15,0
Elixhauser Comorbidity Index	5,0	1,0–17,0	6,0	1,0–17,0

[Description Table 2] Comorbidity scores included all variables, with a lymphoma diagnosis automatically adding 2 points

### Diagnostics, treatments, and side effects

#### Diagnostics (mean proportion within a given year)

In the inpatient setting, a minimum of one computed tomography (CT), magnetic resonance imaging (MRI), and ultrasound (US) were documented for 33%, 9%, and 7% of the FL patients, respectively.

In the outpatient setting, at least one CT was documented for 42% of patients with FL, 19% underwent at least one MRI, and 86% had at least one US. Similarly, in the inpatient setting, 46% of the DLBCL patients received at least one CT diagnosis, 18% an MRI, and 9% an US. In the outpatient setting, 36% of the DLBCL patients received at least one CT, 16% an MRI, and 74% an US.

#### Treatments

Outpatient active substances (ATC L-code, product level) are summarized in the Supplement. Inpatient regimen-level detail is unavailable in OPS; consequently, outpatient and inpatient treatment counts are reported separately and are not additive. The annual distribution of outpatient FL medications at the ATC level, including rituximab, bendamustine, vincristine, doxorubicin, and cyclophosphamide with descending shares, were comparable between 2015 and 2017. The percentage of patients taking obinutuzumab in the outpatient setting increased between 2018 and 2020. (Supplement Table 4) For patients with DLBCL, the distribution of rituximab, vincristin, cyclophosphamide, doxorubicin, and filgrastim/pegfilgrastim, with descending shares, were comparable. The percentage of rituximab decreased slightly over the years. For inpatient medication at the OPS level, the annual distribution changed slightly between 2015 and 2020 (Table 3). For more detailed information see Supplement Tables 4 and 5.

**Table 3:** Overview of the mean number of inpatient treatment procedures performed per year between 2015–2020

	FL		DLBCL	
	**N**	**%**	**N**	**%**
Total prevalent patients	956	100	1,362	100
Non-complex chemotherapy	85	9	265	19
Moderately complex and intensive block chemotherapy	29	3	159	12
High complex and intensive block chemotherapy	8	1	35	3
Other immunotherapy	89	9	310	23
Highly active antiretroviral therapy	0	0	7	1
Radiotherapy	15	2	50	4
CAR T-cell therapy	0	0	< 5	/
Stem cell transplant autologous	7	1	25	2
Stem cell transplant allogeneic	< 5	/	7	1

Results are presented as calendar-year snapshots and reflect average annual values; percentages denote the average annual share of prevalent patients with ≥ 1 corresponding ICD-10 code in that year. For the prevalent FL patients, the following top 5 side effects were documented: reaction to severe stress and anxiety (13%), agranulocytosis and neutropenia (7%), immunodeficiency with predominant antibody deficiency (5%), thrombosis, phlebitis, or thrombophlebitis (5%), and acute renal failure (4%). The DLBCL patients experienced agranulocytosis or neutropenia (16%), reactions to severe stress and anxiety (12%), acute renal failure (9%), aplastic anemia due to cytostatic therapy (8%), and other secondary thrombocytopenia not designated transfusion refractory (7%).

### Healthcare resource utilization (HCRU) and costs in third-party payers perspective

The mean HCRU and associated costs for patients who utilized these resources are presented in Tables 4 and 5.

**Table 4:** Physician visits and hospitalizations mean annual numbers per prevalent patient

		Pat. *n*	Mean	SD	Min	Median	Max
FL	Number of physician consultations (n, ppt)	955	42	27	1	37	290
	Number of hospitalizations	614	2	2	0	1	15
	Inpatient stay (days)	614	21	45	0	3	323
DLBCL	Number of physician consultations	1,355	42	30	0	36	251
	Number of hospitalizations	1,058	3	3	0	2	34
	Inpatient stay (days)	1,058	29	48	0	9	335

In the FL cohort, 64% of patients had ≥ 1 hospitalization on average per calendar year; in the DLBCL cohort, 78%.

The total mean costs averaged €15,258 per patient per year in the FL cohort and €23,455 per patient per year among patients with DLBCL. More details are shown in Table 5.

**Table 5:** Annual total costs for prevalent FL and DLBCL patients in Euro (€)

FL	*n*	mean	median	min	max
2015	837	15,283	6,971	189	202,453
2016	907	15,743	7,251	101	136,487
2017	926	15,683	6,987	0	255,353
2018	1,012	14,938	6,823	287	239,043
2019	1,030	14,846	7,255	147	166,633
2020	1,028	15,054	7,041	187	152,636
**DLBCL**					
2015	1,204	22,309	11,212	0	244,870
2016	1,324	25,046	13,065	116	337,495
2017	1,346	23,906	11,110	0	287,162
2018	1,413	23,130	10,857	0	602,243
2019	1,445	22,235	10,750	104	572,105
2020	1,434	24,101	10,964	0	591,068

In the FL group, the distribution of mean costs per patient was 13% outpatient care, 44% inpatient care (incl. inpatient administered medication), 39% outpatient drug prescriptions, and 3% remedies and aids. Among the DLBCL patients, the distribution was 8% outpatient care, 60% inpatient care, 29% drugs, and 3% remedies and aids.

In total, annual mean costs of €14,566,390 for the FL cohort (*n* = 956 patients) and €31,960,242 for the DLBCL cohort (*n* = 1,361 patients) were documented, respectively. For more details on the total costs, see Supplement Table 6.

### Outcome

#### Documented mortality

The mean annual mortality rate for the FL patients was 5% (*n* = 48 patients). In the DLBCL cohort, 13% patients died annually (*n* = 182).

### Subgroup analysis A: FL patients who underwent stem cell transplantation

In the FL cohort, 38 patients underwent a stem cell transplant during the observation period (auto-SCT: *n* = 27; allo-SCT: *n* = 8). Because of data protection (*n* < 5 patients), no analysis was feasible for remaining patients who received both auto- and allo-SCT.

#### Characteristics and comorbidities

For patients with auto-SCT, the mean age was 59.5 y (SD: 11.68; median: 63 y; range 34–74 y). The sex distribution for this cohort was *n* = 7 (26%) females and *n* = 20 (74%) males. Because of data protection, no detailed information for all patients can be obtained. In terms of comorbidities, the mean Charlson Comorbidity Index (CCI) for autologous transplanted patients was 4.11 (SD: 2.33; median: 3.0; range 2–5). The Elixhauser comorbidity score for auto-SCT patients was 6.78 (SD: 2.65; median: 7; range 3–14).

#### Hospitalization 6, 12, 24 months after transplant

The mean number of inpatient stays for the auto-SCT cohort with a 6-month follow-up(6 M) was: 2.96 (SD: 1.81; median: 3; range 1–9), for the cohort with a 12 month follow-up (12 M): 4.05 (SD: 2.52; median: 3.5; range 1–10), and for patients with a 24 months observation period after SCT (24 M): 6.08 (SD: 3.28; median: 6; range 2–12). The mean number of inpatient days in the 6 M cohort *n* = 57 (SD: 47.12; median: 34; range 21–181); 12 M: *n* = 67 (SD: 69.86; median: 35; range 23–258); 24 M: *n* = 93 (SD: 84.13; median: 49; range 28–239). The mean number of inpatient stays for patients with allo-SCT was only available for patients with 6 months of follow-up after SCT: 3.17 (SD: 0.97; median: 3; range 2–5), with a mean of 121 inpatient days (SD: 45.86; median: 123; range 63–181).

#### Total costs of transplant and 6, 12, and 24 months after

The mean total cost for patients with auto-SCT was €39,729 (SD: 15,076; median: 36,689; range 10,582–82,043) for patients with an observation period of 6 months after SCT. The distribution of this cost was 3% for outpatient care, 88% for inpatient care, 9% for medicines, and 0% for remedies and aids. For patients with 12 M observation period, mean total costs were estimated at €46,270 (SD: 21,936; median: 41,882; range 12,596–106,842) with a distribution of 4% for outpatient care, 79% for inpatient care, 16% for medicines, and 1% for remedies and aids. For patients with 24 M observation period, mean total costs of €57,817 (SD: 34,851; median: 44,758; range 32,521–153,544) were documented. The distribution was 6% for outpatient care, 69% for inpatient care, 25% for medicines, and 0% for remedies and aids.

The mean total cost for allo-SCT patients was €134,983 per patient (SD: 49,058; median: 125,450; range 82,990–202,019) within the 6 months after coding. The distribution of costs was 0% for outpatient care, 86% for inpatient care, 13% for medicines, and 0% for remedies and aids.

Because of the limited number of FL patients, the overall survival probability could not be determined.

### Subgroup analysis B: DLBCL patients who underwent stem cell transplantation

Between 2015 and 2019, *n* = 146 prevalent DLBCL patients received a stem cell transplant. A total of 117 patients received auto-SCT, and 23 patients received allo-SCT. Because of data protection (*n* < 5 patients), no results were shown for patients with auto- & allo-SCT combined.

#### Characteristics and comorbidities

For patients with auto-SCT, the mean age at the time of the SCT procedure was 61 y (SD: 10.2; median: 63 y; range 33–77 y). The sex distribution for the auto-SCT cohort was *n* = 42 (36%) females and *n* = 75 (64%) males. For the *n* = 23 allo-SCT patients, a mean age of 52.7 y (SD: 12.06; median: 55 y; range 24–67 y) was documented. The sex distribution was as follows: *n* = 6 patients (26%) were female, and *n* = 17 (74%) were male. In terms of comorbidities, the mean CCI score for autologous transplanted patients was 6.09 (SD: 2.99; median: 6.0; range 2–13). In the allo-SCT cohort, a score of 4.22 (SD: 1.91; median: 4.0; range 2–9) was documented. The Elixhauser comorbidity score was 8.38 (SD: 2.9; median: 8; range 3–17) for auto-SCT patients, and 6.17 (SD: 2.46; median: 6; range 4–14) for allo-SCT patients.

#### Hospitalization

The mean number of inpatient stays for the auto-SCT patients was 2.47 (SD: 1.44; median: 2; range 0–9) for the 6 M cohort, 3.88 (SD: 3.38; median: 4; range 0–10) for the 12 M cohort, and 6.86 (SD: 4.03; median: 7; range 1–16) for patients with a 24 M observation period after SCT. The mean number of inpatient days in 6 months after transplant was: *n* = 59 (SD: 45.81; median: 39; range 0–207); 12 M: *n* = 80 (SD: 75.60; median: 44; range 17–318); 24 M: *n* = 111 (SD: 109,74; median: 63; range 17–548).

The mean number of inpatient stays for the allo-SCT cohort was 6 M: 2.62 (SD: 1.28; median: 2; range 1–6), 12 M: 6.07 (SD: 3.38; median: 6; range 1–12), and for patients with a 24 M observation period after SCT: 11.11 (SD: 5.33; median: 12; range 2–20). The mean number of inpatient days in 6 months after transplant was *n* = 117 (SD: 59.52; median: 106; range 37–241); 12 M: *n* = 183 (SD: 116.94; median: 144; range 40–350); 24 M: *n* = 234 (SD: 195.53; median: 162; range 40–621).

#### Costs

The mean total cost for patients with auto-SCT was €47,703 (SD: 32,378; median: 37,666; range 10,582–266,580) for patients with a 6-month observation period after SCT. The distribution of this cost was 2% for outpatient care, 90% for inpatient care, 7% for medicines, and 1% for remedies and aids. For patients with 12 M: €56,558 (SD: 45,926; median: 42,064; range 12,596 − 338,009) by a documented distribution of 3% for outpatient care, 82% for inpatient care, 11% for medicines, and 4% for remedies and aids. For patients with a 24 M observation period, mean total costs of €67,022 (SD: 59,175; median: 45,677; range 25,967–373,100) were documented. The distribution was 5% for outpatient care, 77% for inpatient care, 17% for medicines, and 1% for remedies and aids.

The mean total cost for patients with allo-SCT was €123,006 (SD: 49,657; median: 113,496; range 34,142–256,869) for patients with a 6-months observation period after SCT, with a distribution of 1% for outpatient care, 89% for inpatient care, 10% for medicines, and 0% for remedies and aids. For patients with 12 M: €161,662 (SD: 75,266; median: 148,105; range 86,128–294,691) by a documented distribution of 2% for outpatient care, 79% for inpatient care, 18% for medicines and 0% for remedies and aids. For patients with a 24 M observation period, mean total costs of €185,657 (SD: 97,389; median: 189,174; range 88,020–408,537) were documented. The distribution was 3% for outpatient care, 72% for inpatient care, 25% for medicines, and 0% for remedies and aids.

#### Outcomes in terms of documented mortality after transplant

**Fig. 1:**
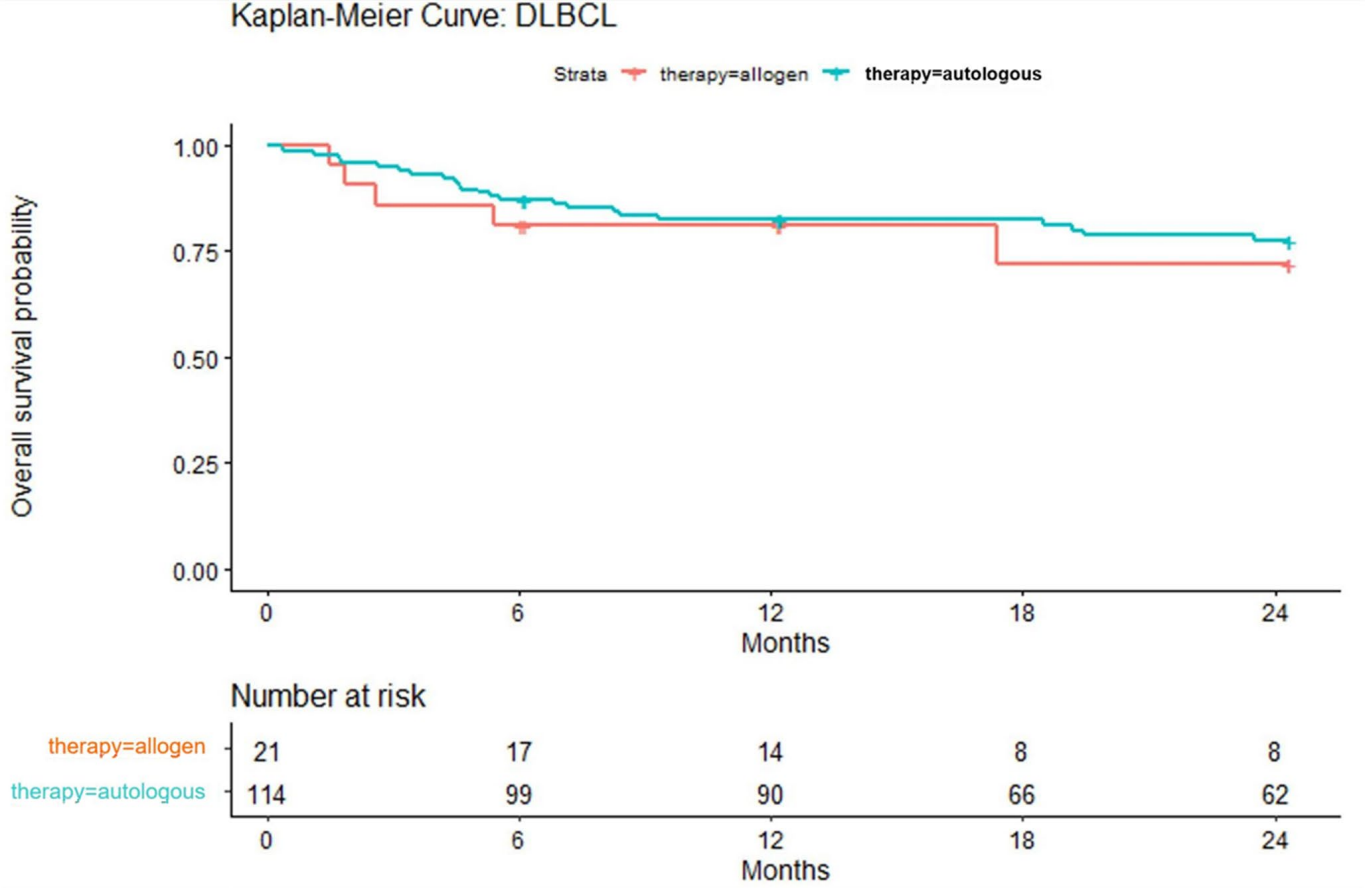
Documented overall survival probability for DLBCL patients after SCT

After 6 months and 12 months post-transplant, the estimated mortality rates in the auto-SCT cohort were 13% (*n* = 15 patients) and 18% (*n* = 20 patients), respectively. In the allo-SCT cohort, n = < 5 patients died. For more details, see Fig. 1. Figure 1 includes only patients with complete follow-up for the shown horizon (6/12/24 months), which may lead to count differences versus totals reported elsewhere.

## DISCUSSION

The findings of this study demonstrate the substantial health economic burden of malignant lymphomas (FL, DLBCL). Between 2015 and 2020, third-party payers spent €87.4 million for *n* = 956 FL patients and €191.8 million for *n* = 1,361 DLBCL patients. To our knowledge, this is likely one of the first comprehensive analyses of German claims data for malignant lymphomas such as FL and DLBCL.

At the European level, annual prevalence rates of 37/100,000 for FL [ICD-10: C82.0, C82.1, C82.2, C82.3, C82.4, C82.5, C82.6, C82.7, C82.9] and 43/100,000 for DLBCL patients [ICD-10 C83.3] were recently published [23]. In our study cohort, the extrapolated prevalence for Germany of the DLBCL cohort was 42.4/100,000, which is comparable to the aforementioned European data. Additionally, there was a trend of increasing prevalence over the observation period. Between 2015 and 2020, the prevalence increased by 23% for FL and 22% for DLBCL. This trend of increasing patient numbers is comparable with German data provided by the “Krebs in Deutschland für 2017/2018” report published by the governmental Robert-Koch Institute [4].

In terms of age distribution, the majority of the patients had a documented age above 60 years. Up to 75% (FL) and 76% (DLBCL) of the patients were in this age group. This observation must be interpreted from a broader view. In the context of a higher mortality risk for patients > 75 y, the low numbers in the eldest age groups in our prevalent cohort are plausible [4]. In terms of sex distribution, the InGef cohort is similar to that of Dürig J et al.: gender is balanced in the FL group (49% men), whereas the proportion of men (58%) was slightly greater in the DLBCL group [24]. This observation is also in line with previous publications [5, 15, 16].

No German publication on comorbidities has been found; therefore, no national comparisons are possible. Yang X. et al. performed a US claims data analysis on *n* = 2,500 DLBCL patients and reported that the mean CCI for prevalent patients was 2.3 (SD: 2.4) [25]. In our study cohort, the CCI for the prevalent DLBCL cohort was 4.8 (SD: 2.7). The CCI is clearly higher may be due to using different methodological approaches (entities not excluded). For FL patients, no publicly available information has been found. In addition to the scores, the individual underlying variables indicate a large patient burden (Supplement Table 3). For example, approximately 30% of all prevalent patients in both entities had depression in our study cohort. In a previous publication in Germany, the depression rate for NHL patients was 22% within 10 years after the index date was communicated [26]. Additionally, the documented side effects indicate the burden for these patients. Here, a clear difference between the two entities was documented. The shares of agranulocytosis and neutropenia, reactions to severe stress and anxiety, and acute renal failure are more than double for DLBCL patients. This observation, as well as the more intense treatment regimens, might be responsible for the documented longer inpatient days in the DLBCL cohort. In addition, the mean numbers of diagnostic procedures shown underscore the high level of resource use, as all patients underwent at least one procedure. It is important to note that our analyses included a specific set of diagnostic-codings, selected as the most relevant in the follicular lymphoma (FL) and diffuse large B-cell lymphoma (DLBCL) contexts, as determined in consultation with the clinical experts. Furthermore, it should be considered that patients often receive a variety of additional interventions that are also captured in billing data beyond the diagnostics analyzed here.

Other results concerning treatment details are generally consistent with the expectations of clinical experts and previous international research. Thus, a substantial percentage of chemotherapy-based treatment approaches for the DLBCL cohort (e.g., R-CHOP) is documented [27]. In contrast, in the FL cohort, 25% of the prevalent patients received rituximab in the outpatient setting only. In addition, the increasing usage of innovative treatments after market approval was documented. After its approval in September 2017, the use of Obinutuzumab for FL patients continuously increased between 2018 and 2020 [28].

When discussing patient burden, hospitalization rates are an additional indicator of previously discussed aspects. The results revealed an annual mean of 20.7 inpatient days in the FL cohort and 29.3 days in the DLBCL cohort. The numbers of hospitalizations were *n* = 2.0 and *n* = 2.9 admissions, respectively. The maximum numbers of *n* = 323 (FL) and *n* = 336 (DLBCL) inpatient days indicate major expenditures of resources and an enormous burden of disease for individual patients. This observation of enormous efforts for a small number of high-risk patients is consistent with a German DLBCL study [29]. In this retrospective analysis of relapsed and refractory DLBCL patients, a mean number of 63 inpatient days (median 66; range 17–123; SD: 36) and 5 admissions (3; 1–12; ±3) were documented in DLBCL patients with > 3 lines of therapy.

However, in our study, the annual costs for inpatient care contributed to the highest shares of total cost for both entities and over the total observation period. A high proportion of inpatient costs was also noted in several international real-world cost analyses of DLBCL and FL [30–32]. A recent German claims data analysis of DLBCL patients documented hospitalization as the main cost driver (71%). The time-unadjusted absolute costs amounted to €59,868, €35,870, and €28,832 during first-line, second-line, and third-line treatments, respectively [33]. Due to methodological differences (e.g., results classified by lines of therapy only), no detailed comparisons were performed. International publications also show comparable results: in a Canadian study of DLBCL patients, the inpatient stay for second-line treatment was the largest cost driver (62%) [31]. In a Japanese claims data analysis, Tsutsué et al. reported that the majority of the overall costs and per-treatment lines were due to inpatient costs (*n* = 6,821) of 47,903.08 USD (SD, 47,497.30; range, 247.43–488,296.86) [30]. A major factor in this context is the choice of therapy. One example of a treatment approach that has an enormous economic impact is stem cell transplantation. In a Canadian study, autologous SCT and hospitalization contributed the most to direct costs for DLBCL patients, corresponding with more than three lines of therapy [34]. We discuss this in the context of the subgroup analysis of SCTs in the following text.

In general, the mean and median numbers of all cost data differ because of the wide range of minimum and maximum numbers. In total, mean costs of €15.4 million are documented per year in the FL cohort, in comparison to €34.6 million in the DLBCL cohort. The mean annual costs from 2017 to 2020 for FL and DLBCL patients in our cohort accounted for a small fraction (0.3% for FL and 0.7% for DLBCL) of the total expenditure on antineoplastic agents in Germany during the same period (approximately €5 billion) [35]. This comparison highlights the relative scale of FL and DLBCL-related costs but also reflects the inclusion of broader expense categories, such as inpatient care, in our analysis, which complicates a direct comparison with national antineoplastic drug expenditures.

In terms of outcomes, we focused on mortality. The mean annual mortality rate for the prevalent FL patients was 5%, and that for the DLBCL cohort was 13%. Since these cohorts are cross-sections of all patients in the corresponding year, only the outcome results of the SCT subgroup are discussed. With respect to the outcomes in the subgroup analysis of stem cell-transplanted patients (Fig. 1), a recent Canadian study on FL patients also demonstrated a potential long-term benefit of SCT in Canada [36]. Owing to the insufficient evidence level based on German data for both entities, no further comparison with national data was possible. However, when discussing treatment and respective outcomes, innovative approaches must be set into perspective, e.g., CAR T-cell therapies [37].

In addition, in the subgroup analysis, the mean total cost in the 12 month after autologous SCT was €46,270 for FL patients and €56,558 for DLBCL patients. The mean cost for DLBCL patients within the 12 months after receiving allo-SCT was €161,662. Mayerhoff et al. reported direct costs of €230,399 per patient (DLBCL/FL allo-SCT) and €107,457 per patient (DLBCL/FL auto-SCT) for Germany. These reported costs are substantially higher because they were summed up in the period of two quarters before and eight quarters after SCT [38]. Moertl et al. reported mean treatment costs per DLBCL patient, with auto-SCT costs of €55,468 and €131,264 for allo-SCT in the clinical setting [29]. Further research and detailed information are needed to frame conventional treatments in the context of innovative treatment approaches, such as CAR T-cell therapies, for which cost ranges between €276,086 and €328,727 [39].

In summary, given the increasing prevalence of hematological malignancies in Germany, comprehensive care for patients can lead to high costs for health systems. Our results can be used as a baseline for future health economic studies in the context of innovative lymphoma therapies in Germany.

Certain factors limit our findings. Nature of claims data. The analyses were based on health claims records, which are collected for billing purposes and not primarily for research reasons. Therefore, all the results depend on the quality of the coding, which may lead to potential over- or underestimations. Because of the high level of data protection in Germany, individual case validation was not possible, and cell counts with *n* < 5 cannot be reported. Moreover, claims lack comprehensive clinical detail (e.g., tumor status, lines of therapy, inpatient medication), socioeconomic variables, and quality-of-life (QoL) information. Regimen detail and sectoral granularity. While OPS procedure codes exist for both inpatient and outpatient services, inpatient systemic therapies are billed via aggregated OPS codes and diagnosis-related groups (DRG) that do not encode individual active substances or dosing. By contrast, the outpatient sector provides prescription-level pharmacy claims at ATC L-code (product) level, which we report in the Supplement. Consequently, inpatient and outpatient treatment figures are not directly additive, and cross-setting regimen-level attribution is not feasible without risking misclassification. Incident ascertainment and prevalence-based design. Prevalence-based design and claims-data constraints. Because incident status cannot be ascertained reliably in anonymized claims (limited look-back, no individual case validation), our cohorts represent cross-sectional, calendar-year snapshots that include both newly diagnosed and longer-term patients. This survivor-enriched, heterogeneous mix may under-represent early post-diagnosis events; results should be interpreted as annual burden among patients under ongoing care, not as incident, near-diagnosis outcomes. Lack of a matched general population comparison. We did not include an age/sex/region-matched reference cohort from the general German population. Consequently, excess risks and costs relative to background rates cannot be inferred, and estimates should not be interpreted as population-level effects. This reflects the scope of our prevalence-based cost-of-illness design. We explicitly caution readers, especially policymakers, accordingly. Interpretation and value of this snapshot. Despite these constraints, the analysis increases transparency for decision-makers by describing real-world healthcare utilization and costs in FL and DLBCL from a payer perspective across inpatient and outpatient settings. It identifies major cost components and utilization patterns and supports planning and evaluation. These descriptive results provide a baseline for future, more granular studies (e.g., incident cohorts or matched comparisons) where feasible.

## CONCLUSION

In summary, in the underlying German health insurance claims database, the number of FL and DLBCL cases continuously increased between 2015 and 2020. The number and length of inpatient stays indicate a high burden for patients and their families. In addition, the costs for SCTs, which is recommended for patients receiving higher lines of therapy, are considerably higher. Future efforts should include linkages to additional data sources, e.g., prospective registries and regional cancer registries, to enable comprehensive information on patient journeys, treatment patterns and subsequent health economic analyses. However, our results from health insurance claims data provide complementary information for initial discussions with payers, administrators, and politicians in the context of access to innovative treatments.

## Supplementary Information

Below is the link to the electronic supplementary material.

Supplementary Material 1 (DOCX 86.7 KB)

## Data Availability

The data used in this study cannot be made available in the article, the supplemental files, or in a public repository due to German data protection laws (Bundesdatenschutzgesetz). To facilitate the replication of results, anonymized data used for this study are stored on a secure drive at the InGef - Institute for Applied Health Research Berlin. Access to the raw data used in this study can only be provided to external parties under the conditions of a cooperation contract and can be accessed upon request, after written approval (info@ingef.de), if required.
