# Supplementary material for: Follicular lymphoma or diffuse large B-cell lymphoma: a population based analysis of epidemiological and health economic aspects in Germany
Source: Ann Hematol. 2025 Sep 2;104(9):4625–35. doi: 10.1007/s00277-025-06592-8 (PMC12552383; doi:10.1007/s00277-025-06592-8)
Supplement: Supplementary file 1 — Supplementary Material 1 (DOCX 86.7 KB) [file 277_2025_6592_MOESM1_ESM.docx]

|  | Year | Mean | SD | Min | Q1 | Median | Q3 | Max |
| --- | --- | --- | --- | --- | --- | --- | --- | --- |
| FL | 2015 | 66 | 13 | 19 | 59 | 68 | 75 | 94 |
|  | 2016 | 67 | 12 | 20 | 59 | 68 | 76 | 95 |
|  | 2017 | 67 | 12 | 18 | 60 | 69 | 76 | 97 |
|  | 2018 | 67 | 13 | 19 | 60 | 69 | 77 | 97 |
|  | 2019 | 68 | 13 | 20 | 60 | 69 | 77 | 98 |
|  | 2020 | 68 | 13 | 21 | 59 | 69 | 77 | 98 |
| DLBCL | 2015 | 68 | 14 | 19 | 59 | 71 | 77 | 93 |
|  | 2016 | 68 | 14 | 18 | 60 | 72 | 78 | 96 |
|  | 2017 | 69 | 13 | 18 | 60 | 71 | 79 | 97 |
|  | 2018 | 69 | 13 | 20 | 61 | 71 | 79 | 98 |
|  | 2019 | 69 | 13 | 18 | 62 | 72 | 79 | 99 |
|  | 2020 | 69 | 14 | 19 | 60 | 71 | 79 | 97 |

**Supplement Table 1: Age distribution for the total prevalent FL and DLBCL cohorts per year**

|  |  | 2015 | | 2016 | | 2017 | | 2018 | | 2019 | | 2020 | |
| --- | --- | --- | --- | --- | --- | --- | --- | --- | --- | --- | --- | --- | --- |
|  | **Sex** | **n** | **%** | **n** | **%** | **n** | **%** | **n** | **%** | **n** | **%** | **n** | **%** |
| FL | Male | 415 | 50 | 449 | 50 | 457 | 49 | 485 | 48 | 515 | 50 | 506 | 49 |
|  | Female | 422 | 50 | 452 | 50 | 470 | 51 | 527 | 52 | 515 | 50 | 522 | 51 |
| DLBCL | Male | 658 | 54 | 746 | 56 | 747 | 55 | 786 | 56 | 804 | 56 | 806 | 56 |
|  | Female | 547 | 45 | 578 | 44 | 608 | 45 | 628 | 44 | 641 | 44 | 631 | 44 |

**Supplement Table 2: Sex distribution for the total prevalent FL and DLBCL cohorts per year**

| Charlson variables (n, %) | FL | |
| --- | --- | --- |
| Any malignancy | 956 | 100 |
| Pulmonary disease | 267 | 28 |
| Diabetes without chronic complication | 223 | 23 |
| Peripheral vascular disease | 222 | 23 |
| Renal disease | 184 | 19 |
| Elixhauser variables (n, %) | **FL** | |
| Lymphoma | 956 | 100 |
| Hypertension | 617 | 64 |
| Depression | 292 | 30 |
| Chronic pulmonary disease | 267 | 28 |
| Cardiac arrhythmias | 227 | 24 |
| Charlson variables (n, %) | **DLBCL** | |
| Any malignancy | 1,362 | 100 |
| Metastatic cancer | 340 | 25 |
| Diabetes without chronic complication | 384 | 28 |
| Pulmonary disease | 343 | 25 |
| Peripheral vascular disease | 460 | 34 |
| Elixhauser variables (n, %) | **DLBCL** | |
| Lymphoma | 1,362 | 100 |
| Hypertension | 923 | 68 |
| Peripheral vascular disease | 460 | 34 |
| Depression | 399 | 29 |
| Cardiac arrhythmias | 398 | 29 |

**Supplement Table 3: Details on the mean annual distribution of comorbidities from prevalent FL and DLBCL cohorts**

|  | 2015 | | | | 2016 | | | | 2017 | | | | 2018 | | | | 2019 | | | | 2020 | | |
| --- | --- | --- | --- | --- | --- | --- | --- | --- | --- | --- | --- | --- | --- | --- | --- | --- | --- | --- | --- | --- | --- | --- | --- |
|  |  | N | % |  | | N | % |  | | N | % |  | | N | % |  | | N | % |  | | N | % |
| FL | N total | 837 | 100 | N total | | 901 | 100 | N total | | 927 | 100 | N total | | 1,012 | 100 | N total | | 1,030 | 100 | N total | | 1,028 | 100 |
|  | Rituximab | 211 | 25 | Rituximab | | 224 | 25 | Rituximab | | 224 | 24 | Rituximab | | 200 | 20 | Rituximab | | 183 | 18 | Rituximab | | 155 | 15 |
|  | Bendamustine | 78 | 9 | Bendamustin | | 73 | 8 | Bendamustin | | 71 | 8 | Bendamustin | | 96 | 9 | Bendamustin | | 84 | 8 | Obinutuzu. | | 81 | 8 |
|  | Vincristine | 49 | 6 | Doxorubicin | | 42 | 5 | Cyclophosph. | | 44 | 5 | Obinutuzu. | | 47 | 5 | Obinutuzu. | | 66 | 6 | Bendamustin | | 74 | 7 |
|  | Doxorubicin | 47 | 6 | Vincristin | | 42 | 5 | Doxorubicin | | 42 | 5 | Cyclophosph. | | 43 | 4 | Doxorubicin | | 46 | 4 | Vincristin | | 38 | 4 |
|  | Cyclophosph. | 46 | 5 | Cyclophosph. | | 41 | 5 | Vincristin | | 40 | 4 | Vincristin | | 41 | 4 | Vincristin | | 45 | 4 | Cyclophosph. | | 33 | 3 |
| DLBCL | N total | 1,205 | 100 | N total | | 1,324 | 100 | N total | | 1,349 | 100 | N total | | 1,414 | 100 | N total | | 1,445 | 100 | N total | | 1,437 | 100 |
|  | Rituximab | 297 | 25 | Rituximab | | 333 | 25 | Rituximab | | 310 | 23 | Rituximab | | 320 | 23 | Rituximab | | 284 | 20 | Rituximab | | 292 | 20 |
|  | Vincristine | 179 | 15 | Cyclophosph. | | 190 | 14 | Vincristin | | 165 | 12 | Cyclophosph. | | 194 | 14 | Cyclophosph | | 192 | 13 | Cyclophosph. | | 183 | 13 |
|  | Cyclophosph. | 175 | 15 | Vincristin | | 189 | 14 | Cyclophosph. | | 163 | 12 | Doxorubicin | | 194 | 14 | Doxorubicin | | 187 | 13 | Vincristin | | 182 | 13 |
|  | Doxorubicin | 171 | 14 | Doxorubicin | | 187 | 14 | Doxorubicin | | 159 | 12 | Vincristin | | 189 | 13 | Vincristin | | 187 | 13 | Doxorubicin | | 182 | 13 |
|  | Filgrastim | 112 | 9 | Pegfilgrastim | | 121 | 9 | Pegfilgrastim | | 112 | 8 | Pegfilgrastim | | 119 | 8 | Pegfilgrastim | | 134 | 9 | Pegfilgrastim | | 147 | 10 |

**Supplement Table 4: Annual TOP-5 distribution of outpatient prescriptions for the prevalent FL and DLBCL cohorts (ATC-L level)**

|  |  | 2015 | | 2016 | | 2017 | | 2018 | | 2019 | | 2020 | |
| --- | --- | --- | --- | --- | --- | --- | --- | --- | --- | --- | --- | --- | --- |
|  |  | N | % | N | % | N | % | N | % | N | % | N | % |
| FL | Total patients | 837 | 100 | 901 | 100 | 927 | 100 | 1,012 | 100 | 1,030 | 100 | 1,028 | 100 |
|  | Non complex chemotherapy | 80 | 10 | 86 | 10 | 103 | 11 | 81 | 8 | 78 | 8 | 84 | 8 |
|  | Moderately complex and intensive block chemotherapy | 28 | 3 | 34 | 4 | 29 | 3 | 32 | 3 | 27 | 3 | 25 | 2 |
|  | High complex and intensive block chemotherapy | 6 | 1 | 12 | 1 | 5 | 1 | <5 | - | 6 | 1 | 10 | 1 |
|  | Other immunotherapy | 82 | 10 | 89 | 10 | 92 | 10 | 88 | 9 | 86 | 8 | 94 | 9 |
|  | Highly active antiretroviral therapy | 0 | 0 | 0 | 0 | 0 | 0 | 0 | 0 | 0 | 0 | 0 | 0 |
|  | Radiotherapy | 13 | 2 | 18 | 2 | 14 | 2 | 18 | 2 | 14 | 1 | 10 | 1 |
|  | Chondrocyte preparations, CAR-T-cells | 0 | 0 | 0 | 0 | 0 | 0 | 0 | 0 | 0 | 0 | 0 | 0 |
|  | Autologous stem cell transplant | 5 | 1 | 9 | 1 | 5 | 1 | 6 | 1 | 5 | 0 | 9 | 1 |
|  | Allogenic stem cell transplant | <5 | - | <5 | - | <5 | - | <5 | - | <5 | - | 0 | 0 |
| DLBCL | Total patients | 1,205 | 100 | 1,324 | 100 | 1,349 | 100 | 1,414 | 100 | 1,445 | 100 | 1,437 | 100 |
|  | Non complex chemotherapy | 239 | 20 | 278 | 21 | 260 | 19 | 284 | 20 | 280 | 19 | 249 | 17 |
|  | Moderately complex and intensive block chemotherapy | 145 | 12 | 174 | 13 | 168 | 12 | 154 | 11 | 158 | 11 | 157 | 11 |
|  | High complex and intensive block chemotherapy | 29 | 2 | 43 | 3 | 36 | 3 | 30 | 2 | 39 | 3 | 34 | 2 |
|  | Other immunotherapy | 253 | 21 | 319 | 24 | 303 | 22 | 320 | 23 | 328 | 23 | 335 | 23 |
|  | Highly active antiretroviral therapy | <5 | - | 7 | 1 | <5 | - | <5 | - | <5 | - | <5 | - |
|  | Radiotherapy | 35 | 3 | 67 | 5 | 54 | 4 | 36 | 3 | 55 | 4 | 52 | 4 |
|  | Chondrocyte preparations, CAR-T-cells | 0 | 0 | 0 | 0 | 0 | 0 | 0 | 0 | <5 | - | 5 | 0 |
|  | Autologous stem cell transplant | 27 | 2 | 24 | 2 | 29 | 2 | 23 | 2 | 21 | 1 | 24 | 2 |
|  | Allogenic stem cell transplant | <5 | - | 10 | 1 | 5 | 0 | 6 | 0 | 6 | 0 | <5 | - |

**Supplement Table 5: Annual distribution of coded procedures of the prevalent FL and DLBCL cohorts (OPS codings)**

|  | Year | Total costs |
| --- | --- | --- |
| FL | 2015 | 12,791,616 |
|  | 2016 | 14,184,069 |
|  | 2017 | 14,538,287 |
|  | 2018 | 15,117,289 |
|  | 2019 | 15,291,429 |
|  | 2020 | 15,475,648 |
| DLBCL | 2015 | 26,881,839 |
|  | 2016 | 33,161,262 |
|  | 2017 | 32,249,340 |
|  | 2018 | 32,705,725 |
|  | 2019 | 32,129,544 |
|  | 2020 | 34,633,740 |

**Supplement Table 6: Annual distribution of total costs in third party payer perspective for the total prevalent FL and DLBCL cohorts in Euro (€**
